# Personal

**Published:** 1891-08

**Authors:** 


### Personal.

Doctor A. L. Hummel and Mr. Charles Roome Parmele have formed a copartnership, under the firm name of Hummel & Parmele, for the purpose of carrying on a medical journal advertising agency. The New York office of the firm is at 19 Park place, New York City, and the Philadelphia office at 612 Drexel building, Philadelphia. These gentlemen are both well and favorably known to the medical profession of this country, and we bespeak for them a generous patronage. They are reliable, honest, and energetic, and we wish the new firm a prosperous career.

Dr. Edwin Walker, of Evansville, was elected President of the Indiana State Medical Society, at its meeting in Indianapolis, June 10, 1891,— an honor to him, to his city, and to the young men who supported him so faithfully. He is by many years the youngest man elected to the highest office in the power of the medical profession of Indiana to give. The young men will feel it incumbent on themselves to see that the next year in the history of the Indiana State Medical Society be a memorable one, and that the meeting exceed all others. They will not fail.

Dr. Jos. Eastman, of Indianapolis, has received the degree of LL. D. from the Wabash College, of Indiana. The doctor is a genial gentleman of deservedly high standing in the profession, and we congratulate him on this high mark of esteem bestowed upon him by those who are in a position to know his worth.—*Lancet-Clinic.*

